# Applying the robust adaptation planning (RAP) framework to Ghana’s agricultural climate change adaptation regime

**DOI:** 10.1007/s11625-017-0462-0

**Published:** 2017-08-19

**Authors:** Abrar S. Chaudhury, Thomas F. Thornton, Ariella Helfgott, Chase Sova

**Affiliations:** 1 Environmental Change Institute, University of Oxford, Oxford, UK; 2 CGIAR Research Program on Climate Change, Agriculture and Food Security (CCAFS), Wageningen, The Netherlands

**Keywords:** Adaptation planning, Agriculture, Robust action, Network analysis, Climate change, Ghana

## Abstract

This paper introduces a five-step framework, namely the Robust Adaptation Planning (RAP) framework, to plan and respond to the ‘grand challenge’ of climate change. RAP combines, under a unified framework, elements from robust action, participatory planning and network theory to capture the different motives, perception, and roles of actors that are important for climate change adaptation. RAP leverages existing structures and networks and involves diverse actors to plan, sequence and time strategies across multiple levels (i.e. from local to national). Actors identify adaptation interventions and important actor relations to develop wide networks, highlighting potential pathways for connecting action from central policy to local implementation (and vice versa). Comparing these proposed participatory structures with existing structures reveals actors deemed important for delivering adaptation, as well as gaps and overlaps in their relations. The end result is a robust plan covering many perspectives and local realities for both relieving immediate and adapting to longer-term consequences of climate change. We applied the RAP framework in Ghana’s agricultural climate change adaptation regime to demonstrate its usefulness as a means of planning adaptation interventions in a climate-vulnerable, multi-actor and multi-level setting. The application of the RAP framework in this paper highlights how it can: (1) visualise the adaptation space (and its different components), and reduce the complexity of implementing adaptation responses; (2) offer a shared space to actors from all administrative levels to think and create collective narratives for adaptation without demanding explicit consensus and; (3) identify key actors and actions through a collaborative planning process, and allocate responsibility for the smooth delivery of adaptation interventions.

**Electronic supplementary material:**

The online version of this article (doi:10.1007/s11625-017-0462-0) contains supplementary material, which is available to authorized users.

## Introduction

Climate change has emerged as one of the greatest threats facing humanity in the 21st century. Such is the scale of the problem that it is labelled a ‘grand challenge’ or a ‘super wicked problem’ with no obvious solution and no single actor capable of offering comprehensive resources or knowledge to tackle it (Ferraro et al. 2015; Lazarus 2008). Sub-Saharan Africa is already affected by climate change and future impacts are expected to be substantial (IPCC 2014). This is especially pertinent to agriculture that supports millions of rural households across the continent (FAO 2012), and is the key economic driver of many African economies.

Changes in rainfall patterns and higher temperatures in the region can reduce crop yields and increase the risk of crop failure (Nelson et al. 2009). Furthermore the high reliance on natural resources, limited ability to adapt financially and institutionally, low per capita Gross Domestic Product (GDP), poverty, and a lack of safety nets mean that climate change poses a considerable challenge in sub-Saharan Africa, especially for rural communities and the agricultural systems they depend upon (Schlenker and Lobell 2010).

Unprecedented and coordinated responses are required of many disparate actors, from national governments, and international organisations to civil society and individual researchers/practitioners in order to help vulnerable populations adapt to the adverse impacts of climate change (Bryson 2004; Ostrom 2010a). Invariably, the objectives, motives and actions of these actors are incompatible or misaligned with the goal of effective adaptation to climate change, leading to ill-defined and weak responses (Chaudhury et al. 2016b; Mermet 2011). Capturing the different motives, perception, and roles of these many actors under a unified framework is a major challenge (Etzion et al. 2015), but necessary to develop effective climate change adaptation strategies and plans for agricultural systems.

Identifying strategies and responses that are practical, sound and meet the actors’ varied interests requires careful and flexible planning. This is challenging because most adaptation measures are usually planned and implemented by public actors within national structures that are rigidly bureaucratic, incremental, and often underemphasise the role of many other actors (Adger et al. 2005; Scott 1998). Added to this complexity is the lack of consensus on defining adaptation (Adger et al. 2003; Füssel 2007; Smit et al. 2000) or characterising the scales and levels involved in tackling adaptation (e.g. temporal and spatial scales) (Cash and Moser 2000; Gibson et al. 2000).

Effective responses to climate change require both the immediate relief from extreme events, as well as the development of longer-term plans that match the roles and capacities of actors with the appropriate scales and levels of the overall challenge (Gibson et al. 2000). For agriculture, this may entail short-term measures such as securing heat and drought tolerant inputs for local farmers that experience uncertain rains during the planting season (Tambo and Abdoulaye 2012), as well as longer-term improvements in farming practices through crop diversification, among others (Lin 2011).

The published literature recognises that good collaboration among affected stakeholders is crucial for effective adaptation action (Bodin and Crona 2009; Cassidy and Barnes 2012; Folke et al. 2005; Newman and Dale 2005). However, there is little information about suitable methods and frameworks for coordinating the actions of different actors, and developing responses to climate change across multiple levels of time, space, and socio-political organisation. Much of the recent work on adaptation has focused either at broad policy and governance levels (Sandström and Rova 2009; Stein et al. 2011; Vignola et al. 2013), or at highly context-specific local levels through local case studies (Reid et al. 2007; Schipper et al. 2014). Actually adaptation studies have seldom focused across levels (Sova et al. 2015, 2016). Even when actors and adaptation actions are identified, it has been challenging to organise the necessary communication and implementation across levels and scales (Hajer and Wagenaar 2003; Hill and Engle 2013).

To meet this gap, we introduce a five-step framework, namely Robust Adaptation Planning (RAP). In particular the scope of RAP is to assist the planning of appropriate interventions to respond to the wicked and grand sustainability challenge of climate change adaptation. RAP builds on the ‘robust action’ approach that is defined as “noncommittal actions that keep future lines of action open in strategic contexts” (Padgett and Powell 2012, p. 24). Robust action offers a suitable base for the RAP framework as it encourages responses to immediate climate change impacts without losing sight of long-term trends and emerging needs (Ferraro et al. 2015; Nair and Howlett 2016; Padgett and Ansell 1993). RAP leverages existing institutional structures and stakeholder networks by using diverse actors to plan, sequence and time adequate strategies across many levels to reduce risks of failure and avoid duplication (Adner 2006).

At the heart of RAP lies a participatory approach that promotes a collective response from actors, without requiring explicit consensus on the semantics of adaptation. Actors identify adaptation interventions and important relations to develop wide networks, highlighting potential pathways for connecting central policy to local implementation and vice versa. By comparing these proposed participatory structures with those actually existing on the ground, RAP can identify actors that are important for delivering effective climate change adaptation, as well as the gaps and overlaps in actor relations/linkages. This can result in a robust adaptation plan that can cover many perspectives and local realities.

To demonstrate the utility of RAP for adaptation in climate-vulnerable, multi-actor and multi-level contexts, we piloted it in agricultural settings of Ghana dominated by subsistence agriculture smallholders.^1^ Ghana offers three appealing features for the RAP application: a high vulnerability to climate change, a developing country setting and a high reliance on agriculture (Government of Ghana 2010; McSweeney et al. 2010). As part of the RAP process selected actors from across Ghana developed plans collaboratively to implement priority adaptation interventions for a small vulnerable agricultural community.

The paper begins with a theoretical introduction to robust adaptation planning for climate change. We do not provide a comprehensive literature review of the disciplines underpinning RAP since these are mature, well researched and widely accepted (e.g. Ferraro et al. 2015; Mikkelsen 2005; Scott and Carrington 2011). Instead, we discuss aspects that are important for developing the RAP approach. We then outline the five-step RAP framework and the methodology adopted for the specific application in Ghana. We present the main findings from the application, assess the usefulness of the RAP framework in a multi-actor and multi-level setting, and outline future opportunities and recommendations for its application.

## Methodology

### Theoretical foundations of robust adaptation planning (RAP)

#### Robust action and participatory planning

The objective of RAP framework is to develop robust plans to balance the need for short-term responses and adapting to the long-term consequences of climate change. Unlike traditional organisational plans that emphasise clear objectives and steps to achieve specific goals (Bolman and Deal 2013; Daft et al. 2010); RAP encompasses strategies that incorporate multiple interpretations of climate change and adaptation, without demanding a unified definition of adaptation (Lempert and Schlesinger 2000). In particular, we propose Ferraro et al. (2015) robust action ‘multi-vocal inscription’ and the ‘participatory architecture’ strategies that promote structures and rules for interaction among many actors across levels, which creatively combine opposing views on the problems of defining adaptation and selecting responses. Multi-vocal inscription, where a single action can be inscribed and interpreted from many perspectives (Padgett and Ansell 1993), offers an appropriate strategy to sustain engagement even when consensus is unlikely.

At the same time, participatory architecture provides a means of engendering and sustaining the involvement of diverse actors to foster long-term engagement. Participation helps overcome the bias of policy-driven or locally–focused approaches by providing space to express diverse opinions, which makes the overall approach more deliberative, effective, and efficient (Chaudhury et al. 2016a; Mikkelsen 2005). This is important because adaptation action involves so many people, scales, and levels, where each may have different perspectives, biases, and interests related to the adaptation processes. Forcing a strict definition of adaptation actions is problematic and may disorientate or unnecessarily constrain people when application is unclear. Robust action allows coordination between different actors by developing focused, yet broad, approaches that are applicable in various plausible scenarios (Vervoort et al. 2014), without requiring explicit consensus.

However, scaling the participatory framework to feed into policy, without losing the quality of participation is challenging. Other criticisms of participatory approaches include methodological weakness of the participatory tools, technical limitations of participation, elite capture of the process and limited impact of actions (Cooke and Kothari 2001; Mosse 2004). RAP overcomes some of these limitations by involving a wide variety of actors in the design, help to articulate collective needs, create meaning and value for collective actions, and minimise blind spots (Brown 2009; Chaudhury et al. 2016a).

#### Network theory

Networks are gaining attention as an effective means of capturing different forms of participation and relations under one umbrella (Borgatti et al. 2009; Hanneman et al. 2005; Ibarra and Andrews 1993; Scott and Carrington 2011; Wasserman 1994). Network analysis is an appropriate building block for RAP as it offers both a theoretical framework for understanding the causes and consequences of relationships and a methodological framework for visualizing and measuring these relationships (Marsden 1990; McCarty and Bernard 2003). Networks can provide essential resources, knowledge, and information that otherwise are not available within a single actor (Bodin et al. 2006; Newman and Dale 2005). They can also influence and spread effective agricultural practices, thus enhancing widespread action for adaptation. Networks help identify key actors, gatekeepers and bridges (Burt 2002; Granovetter 1973) in the adaptation structure who, with the right support, can offer valuable contributions. Detailed network maps are created from the interactions of actors for one or more types of relationships, which are then measured applying network metrics (Borgatti et al. 2009; Wasserman 1994). The mapping and measurement of networks are important to understand and visualise the overall structure and the different pathways linking all levels within the adaptation regime.

### Research approach of the robust adaptation planning (RAP) framework

**Fig. 1:**
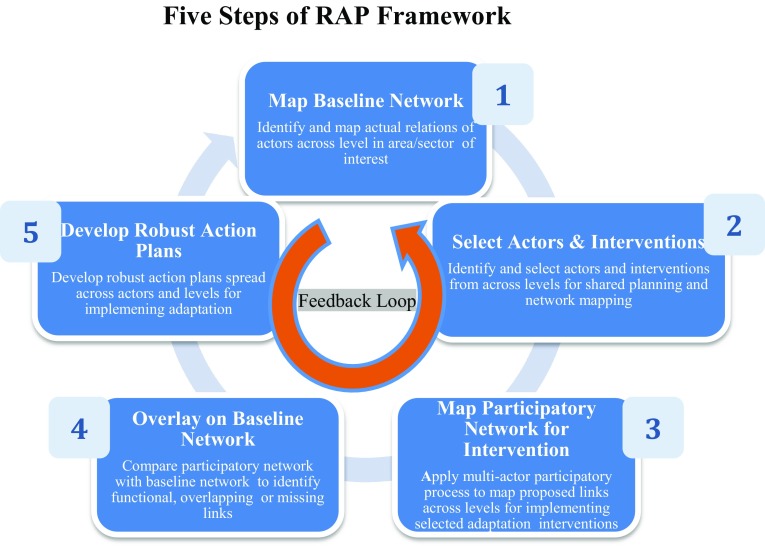
RAP five-step framework

The RAP framework follows a five-step process (Fig. 1) as discussed below. These five steps occur sequentially and inform one another through feedback loops. The RAP framework may require several iterations to find the appropriate balance between actors and relations and, in turn, robust implementation. The RAP analysis works with planned interventions, whose benefits are uncertain. This analysis is typically initiated and undertaken by the RAP project leader (either a researcher or a representative from the public or private sector) who acts as a process facilitator or knowledge broker (Wittmayer and Schäpke 2014). The invited workshop participants support the RAP leader in developing the proposed participatory mapping and actions plans.

Step 1 entails the mapping of the baseline actor network. The RAP project leader is responsible for mapping the actual relations of actors active or affected by adaptation. The network can be mapped through several approaches, such as backward mapping that builds the analysis from the lowest level of implementation or forward mapping that is driven by policy objectives (Elmore 1979). The network may be a complete network (i.e. include all actors in a network) or an egocentric network (i.e. focus on relations of a single actor) (Hanneman and Riddle 2011). The relations may be single or weighted, and represent multiple interactions such as for knowledge, resources, or services facilitating adaptation. The objective of this step is to produce a baseline that depicts the wider network based on the actual relations of actors across multiple levels, yet maintains a direct pathway to the focus of interest (e.g. a community, region or an economic sector such as agriculture or forestry). The national level generally assumes the role of formulating policies and implementation strategies based on national priorities, while the local level focuses on implementing the specific policies and strategies because it is there that climate change impacts are felt and most effectively countered (Ostrom 2010b). The baseline network should, therefore, match the level and scope of analysis for effective action.

Step 2 entails the selection of the participating actors and adaptation interventions that frame the process. Actors identified during the baseline network (Step 1) are invited to develop the participatory network (Step 3) for selected interventions within an area of interest. The participants for RAP are typically chosen from actors most interested in (or affected by) the adaptation regime (Green 2002), or where their participation is necessary to ensure successful implementation of the adaptation initiatives. These can be individuals, groups or organisations. Independent experts and decision makers can also be included to add breadth. Selecting participants is an imperfect and recursive process that will vary with location and scope of the analysis. It is important to have a broad and balanced representation of actors from all levels (i.e. from local community to national policy), categories (e.g. state, non-state, community, research) and network (i.e. central or peripheral actors) that can usefully contribute. An unbiased selection criterion that is communicated clearly to the participants makes the process more inclusive and transparent (Biernacki and Waldorf 1981; Goodman 1961). It also respects the right of actors not to participate, while participants can nominate representatives (e.g. senior personnel or knowledge expert) or nominate others that can contribute meaningfully. It is also important to communicate clearly to the participants (in person if practical) before the RAP workshop (Step 3), the objectives, goals and steps of the framework. This helps gain necessary information, build consensus, and address some of the questions about legitimacy, motivation, representation, and credibility of the process and its participants.

While the RAP framework can be applied to any adaptation intervention, it is important to select those that are important to all actors, to secure strong buy-in. One approach is to focus on issues originating from the ground. Established participatory techniques and methods can be used to enable people to define the actions they require (Chambers 1992; Chaudhury et al. 2016a; Mcgee 2002). Alternatively, policy-based actions can be selected from broader national development goals or sectorial priorities. The RAP leader may prepare (with support from experts) and present to the participants a long list of existing adaptation interventions covering local challenges and policy goals. The participants can select (and if needed) modify, combine or develop from scratch adaptation interventions for RAP. While this paper demonstrates the applicability of the RAP framework for any selected intervention, Step 2 can also help refine standard interventions based on the collective input of the participants.

Step 3 involves the mapping of the participatory network. This step begins with a participatory workshop comprising those actors invited in Step 2. The workshop offers the participants a dedicated space to develop detailed, hypothetical network maps of actors and relations collectively for the interventions selected during Step 2. Together the participants propose the processes, actions, actors and links needed for implementing the selected adaptation actions.

First, the workshop recognises the different roles of each participant. This helps promote a deeper appreciation of each actor’s roles within the network and helps overcome any culture of blame (e.g. farmers are lazy, officials are corrupt). The participants draw diagrams and rich pictures (Lewis 1992; Monk and Howard 1998) of the adaptation space on a sheet of paper. These can include institutions, organisations, actors and other elements, as a way of gathering and representing information about complex situations. Drawings can both evoke and record insight into a situation and are useful to allow groups to explore their subconscious and conflicted understanding of the adaptation challenge (Bell and Morse 2013). The workshop produces detailed layered maps that show the actors and activities with respect to the selected intervention and provides a visual basis for exploring connections and disconnections. Participants mark lines on the map representing proposed links between actors, such as how knowledge and resources should flow. This allows them to capture and acknowledge the complexity and perspectives within the adaptation system. The output of this step, is thus produced by the participants, and is a multi-level network map of the links needed between actors to implement the selected interventions.

Step 4 overlays the participatory network map produced during Step 3 onto the baseline network produced during Step 1. This procedure shows the implementation pathways based on actual relations rather than those assumed to be optimal for implementation. For the successful implementation of the identified interventions this step identifies actors and their links that are functional, overlapping or missing. These are colour coded to identify the pathways (e.g. green for functional links and red for missing links). The comparison is intended to facilitate cross-level analysis and demonstrate flows of knowledge and resources, thereby identifying links to work with and build from. Identifying missing but important links offers opportunities to decide what is required to establish these links and ensure that information, knowledge, and resources get to where they are needed the most. This process helps create new insights by sharing ideas across all levels and instils hope and optimism about what can be achieved simply by improving communication.

Step 5 undertakes the development of the robust action plans. In this final step, the participants produce detailed action plans using relational data from the previous steps to implement the chosen intervention. The main objective of Step 5 is to translate the needs identified through the RAP process into a robust plan for implementation. Action plans are broken-down into detailed, concrete steps that define tasks and roles. Thus they create a sense of ownership and responsibility within a complex process. The plans address strategies for establishing links between disconnected actors to improve cross-level collaboration while strengthening existing links. The primary outcome of this step is a range of response options that improves alignment and integration of adaptation initiatives, and provides ownership for actors at every level. Participants are helped through workshop facilitation to consider actions and links between levels. They also identify suitable conditions for these links to function effectively, thereby producing cross-level integrated response pathways. This helps to uncover gaps, overlaps, trade-offs and synergies between actions of actors operating at different levels. This process leaves participants with a clear vision of the implementation process by improving alignment and integration. The plans incorporate steps to distribute responsibility amongst the actors. Focal actors or champions are assigned by the workshop participants to lead the implementation. Timelines are agreed and roles of other actors are defined, setting responsibility for action.

The action plans for the selected interventions can be viewed against different social and economic scenarios (Vervoort et al. 2014). This can facilitate testing their robustness and covering uncertainties, along with a portfolio of actions that can be referred to as circumstances change. This ensures that decision-makers are aware that plans are sensitive to future uncertainties and keep future lines of action open.

### Study site

The RAP framework was developed as part of Systemic Integrated Adaptation’ (SIA) project within the CGIAR Research Program on Climate Change, Agriculture, and Food Security (CCAFS) and is housed in Oxford’s Environmental Change Institute (ECI).

We piloted the RAP framework in the agricultural adaptation regime of Ghana. Adapting to climate change in agriculture is a recognised goal for Ghana, as it is confronted by increasingly variable rains, floods and droughts, which threaten rural livelihoods and food security (Government of Ghana 2015; McSweeney et al. 2010; Stanturf et al. 2011).

In particular, for the RAP application we selected the Lawra District in Upper West Ghana because it is home to our extensive study of the regions and several CCAFS baseline study sites (Chaudhury et al. 2017; Naab et al. 2011; Sova et al. 2016). Farming is particularly challenging in this part of Ghana due to the low socioeconomic development and adverse environmental and climatic conditions, (Antwi-Agyei et al. 2012; Etwire et al. 2013).

According to the official district records, Lawra district has 120 villages with an average size of 54 households and a median of 43 households. We shortlisted and visited eight villages within Lawra to evaluate the logistics, village size and social dynamics (a representative population), livelihoods and agricultural production patterns and community desire to be involved in research. Orbili Village, with its population of 156 adults in 58 rural households, offers a representative and appropriate focal point from which to build the multi-level RAP process.

**Fig. 2:**
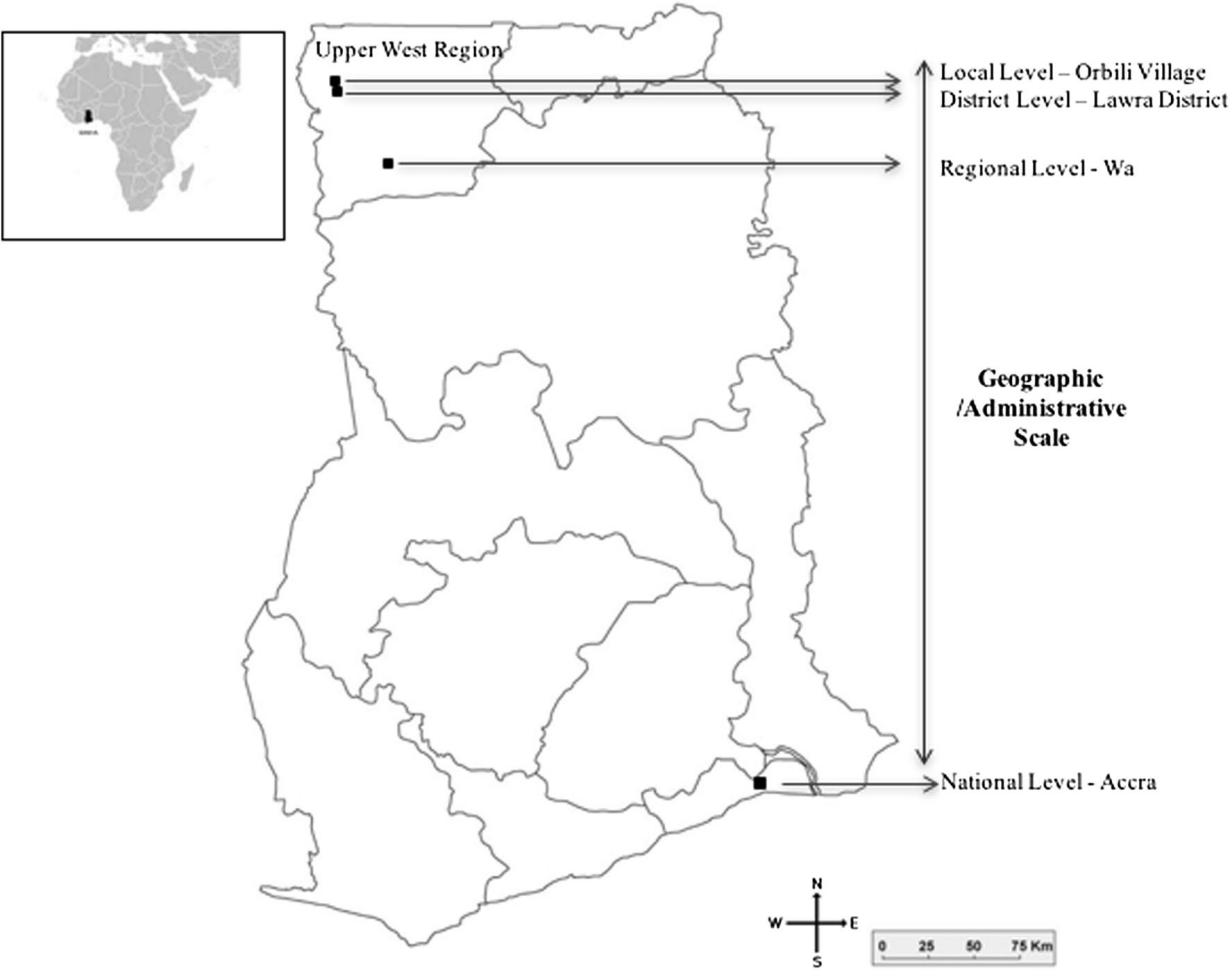
Map of Ghana showing Upper West Region Source: Original Ghana map from: www.mapsoftheworld.com

We must note that as the analysis in this paper is based on a single case study, care must be taken in generalising the results. As this paper attempts to highlight the RAP methodology and its functionality, rather than focus on the utility and desirability of the selected interventions, a single case village offers an appropriate context for the application of RAP. Figure 2 shows the administrative/geographic scale spread across the local, district, regional, and national levels, as mapped from Orbili.

### Data collection and analysis

We adopted a mixed-methods research approach (Silverman 2013) that combined qualitative and network analysis supported by fieldwork. Table 1 summarises the data, methods and deliverables for each step of the RAP application process in Orbili. We collected baseline relational and qualitative data through workshops, surveys, network mapping and detailed semi-structured interviews over several months of field research in 2013–2014. To develop the baseline over 150 interviews were conducted with key national, sub-national, local and community-level actors engaged in climate change adaptation (Step 1).

**Table 1:** Summary of the RAP pilot application

RAP steps	Duration	Data collected	Analytical methods	Deliverables
Step 1 Map baseline network	3–6 weeks for network data collection. 2 weeks for network data analysis	Relational data of key actors from local to national levels, based on the egocentric Orbili	Interaction protocol to capture egocentric network, actor interviews, Gephi social network analysis software	Baseline network map of actors across Ghana based on their multiple relationships (Fig. 3, and Table S1 in Supplementary Electronic Material)
Step 2 Select actors and interventions	2–3 weeks for producing the suite of interventions. 4–6 weeks for organising the participatory workshop and selecting/inviting participants	On-the-ground challenges from local community. Expert input to develop interventions to meet the challenges	Community diagnostic workshop at Orbili, expert consultations, content analysis, personal visits to potential workshop participants	Suite of adaptation interventions. Selection of 40 participants for the Accra workshop (Supplementary Electronic Material)
Step 3 Map participatory network	3-day participatory workshop in Accra	Proposed processes, actions and links between actors to implement the interventions	Participatory approaches, rich pictures and diagrams, network mapping of relations	Multi-level network map of the links between actors necessary for implementing the selected interventions of AIMS and SAI (Fig. 4)
Step 4 Overlay on baseline network	3-day participatory workshop	Participatory network map from Step 3 and baseline relational maps from Step 1	Network analysis (network metrics)	Colour-coded network map showing functional (red) and missing (green) links for selected adaptation interventions (Fig. 5; Table 2)
Step 5 Develop robust action plans	3-day participatory workshop	Identification of action tasks and allocation of roles, using data from Steps 1 to 4	Participatory planning approach	Detailed action plans (divided in tasks and roles) to establish disconnected (and strengthen) existing links for implementing AIMS and SAI interventions (Supplementary Electronic Material)

We applied an egocentric approach (Hanneman and Riddle 2011; Marin and Wellman 2011) to capture the baseline network (Step 1). The network is mapped around a particular node, known as an ego, which in our case is Orbili village (Fig. 2). We developed a detailed interaction protocol to capture primary relational data of the actors. In particular the interaction protocol captured four main aspects of actor relations, namely length, frequency, type, and usefulness.^2^

Instead of constructing the network based solely on data from the ego, we build it through collecting data across different levels. In particular we started with the 58 households in Orbili, and we progressively mapped the network for more distant actors across different administrative layers, spanning from the community, district, and region to the national level. Through this iterative process, we obtained a wide and representative network of actors (from local to national) who were involved in agricultural adaptation, yet maintained direct pathways to Orbili [refer to Supplementary Electronic Material for actors in baseline network (Table S1)]. This corresponds well with the multi-actor and multi-level approach of the RAP framework.

We used Gephi version 0.82 (https://gephi.org), an open-source Social Network Analysis (SNA) software, to process the data. We also adopted a multi-relational approach, which is a valuable analytical area for SNA (Wellman and Wortley 1990). The analysis elicited four types of interactions among the actors that collectively help to assess capacity for adaptation: (1) flow of resources, (2) knowledge transfer, (3) service exchange, and (4) social connections. For instance, flow of resources can lead to improved agricultural productivity and diversity creating a virtuous cycle of income and investment (Deressa et al. 2009). Knowledge transfer can enable the exchange of traditional and expert ideas between organisations that address climatic risks, and encourage suitable adaptive practices (Folke et al. 2002). Social connections may not directly influence adaptation, but it links actors with others who are important resources.

We then assigned weights to the relations based on their multiplicity between actors and the effectiveness of each (as scored by actors) to identify central actors using Eq. 1.

1 $$\text{W} = \sum R \cdot \times \cdot E$$

where W = weight of the relation, R = type of relations, i.e. knowledge, resources, services exchange and connections (where, 1 = relation 0 = no relation), and E = effectiveness of relation (scale of 1–3, where 1 = least effective and 3 = most effective).

We conducted a three-day community diagnostic meeting in Orbili comprising 26 men and 36 women that represented each of the 58 households (voluntary participation, followed with interviews). This diagnostic meeting helped us understand the local environment, development challenges and the responses to climatic and environmental change adopted in agriculture. With input from experts across many levels and content analysis, a suite of adaptation interventions was collated for subsequent RAP application, based on actual and prioritised challenges faced by locals (see next section).

We organised a three-day workshop in Accra for Steps 2–5 that brought together 40 participants from across the different administrative levels as identified through the baseline mapping. Workshop participants developed the participatory network, which culminated in robust adaptation plans. The participants included Orbili farmers, district officials, NGOs, regional coordinators, private sector, international institutions and government ministries, amongst others [refer to Supplementary Electronic Material for a list of participants (Table S2) and a visual agenda for the workshop (Figure S3)]. We included independent experts and decision makers to add breadth to the workshop participants.

Before the workshop, we visited potential participants to brief them about the RAP methodology and the expected outputs of the adaptation action plans. In Orbili, we encouraged households to self-select participants to gain ownership of the process. In particular, we focused on a balanced representation from each administration level and stakeholder category (refer to Figure S1–S2, Supplementary Electronic Material), while also encouraging gender balance. The workshop structure and specific tools applied are covered in more detail in Helfgott et al. (2014).

### Adaptation interventions

As mentioned above, Step 3 of the RAP procedure entails the selection of the adaptation interventions that frame the participatory process. In this paper, the workshop participants selected two adaptation interventions for RAP application (see Results for more information about the selection process). The selected interventions are Agriculture Information Management System (AIMS) and Sustainable Agriculture Inputs (SAI).

These interventions focus directly on the key challenges identified by the local community in the diagnostic workshop (see previous section), address issues of adaptation of agricultural systems to climate change, and require cross-level effort through many actors. Hence, they were considered suitable by the workshop participants for the RAP framework application.

The AIMS was developed by the RAP research team (with experts for the RAP testing) and its focus is to produce a comprehensive agricultural extension policy and accompanying legislative instrument to close the gap between the current extension support offered by the government, and the needs of local farmers. AIMS disseminates to local farmers (and for training extension staff) information on appropriate adaptation strategies and technologies in the form of workshops, field demonstrations, trainings and literature. This would enable the exchange of traditional and expert ideas between local communities and external actors to address climate challenges and encourage suitable adaptive practices. The head of an NGO aptly pointed out that “*whenever a new (agricultural) technology is discovered, it needs to be communicated to the farmer. Capacity building for program officers, technical, agricultural extension officers, is key and that is where the bulk of the funding should be going”* (Personal communication 2013). Similarly, an extension officer attributed low extension support on the shortage of funds suggesting that “*as an extension worker, you get frustrated…because you do not have the resources for your plans. So when you see the farmer you start avoiding him*” (Personal communication 2013).

The SAI was also developed by the RAP research team with experts for the RAP testing. Its aim is to promote the sustainable and informed use of agricultural inputs to improve productivity and crop diversity. This can create a virtuous cycle of income and investment for farmers. Within SAI, the main focus is on the sustainable use and fair allocation of fertilisers to farmers, because of the frequent shortages in the supply chain across regions and districts of Ghana. SAI envisions improving transparency, accountability, and coordination within the national distribution system (through district and regional directors) for the effective management and timely import/manufacture of fertiliser stock to meet local demand. The key challenge of SAI was aptly highlighted by a representative of a local civil society organisation who stated that farmers “*didn’t get fertiliser last year because it was sitting in a warehouse somewhere. So all the farmers were waiting for their fertiliser and by the time it came the growing season was over*” (personal communication 2013).

## Results

### Establishing relations between actors

**Fig. 3:**
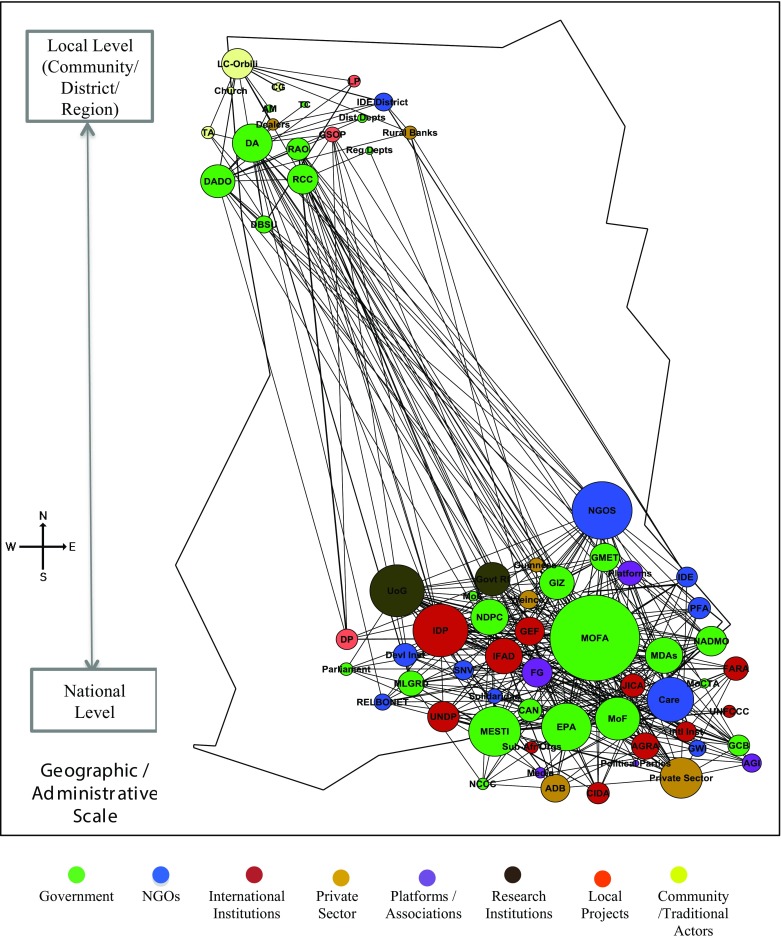
Baseline network map: Undirected network map of adaptation actors: Nodes on the map represent the 66 actors in the network from local to national actors. The edges between the actors are weighted to represent multiple relations and strength of interactions between actors. The node size of actors is proportional to the number of weighted edges connected to the node. Refer to Table S1 in the Supplementary Electronic Material for names of actors and more details

Figure 3 illustrates the baseline network map of actors (colour coded by actor category) active in agriculture adaptation spread across all administrative levels as drawn following an egocentric approach (with Orbili village being the ego) (Step 1). In total, we mapped 66 actors connected through 350 direct relations and 3054 weighted relations. The network is sparsely connected with a density of 0.16 (i.e. the proportion of all possible relations that are actually present).

On the other hand the overall network is diverse with multiple categories of actors being represented (Fig. 3). However, government represents over a third of all actors in the network. This is to be expected as the implementation of formal agricultural activities in Ghana is the responsibility of government agencies, and is thus promoted through the official administration structures. We also observe a high concentration of national actors representing 72% of the network, as opposed to 28% of local level actors at the level of the community, district, and region. This is again not surprising as national actors have a broader interest in climate change adaptation across the country but nonetheless highlights the skewed structure of the network. Although the network is a single connected component because of the egocentric approach adopted, the diameter of the network (i.e. the shortest path between two distant actors) is 5. This highlights the significant separation between local and national actors, with all the ramification it can have for the effective implementation of plans and strategies.

Overall, the Ministry of Food and Agriculture (MOFA) is the apex organisation for developing agricultural policy in Ghana. It assumes the central role in the network linking many actors across levels that are otherwise weakly connected. Similarly at the local level, the District Assembly (DA) and the District Agriculture Department Office (DADO) connect Orbili and other local actors, to the wider network.

We also see a strong presence of NGOs (representing a fifth of actors) because they play an active role in climate projects and ancillary services. International institutions, climate platforms, community actors and the private sector are also visible in the network. Each category of actors has its distinct organisational characteristics and operational structures. This influences how climate change adaptation is framed, activities are prioritized and relations established across all levels [refer to Supplementary Electronic Material for details of actors in baseline network (Table S1)].

### Selecting adaptation interventions

The main challenges identified by households of Orbili village were low income, low crop yield, poor access to agriculture inputs and implements and variable rainfall. With input from experts across many levels and content analysis, the research team collated a suite of adaptation interventions for Orbili village. Based on this understanding of the local context the RAP facilitator team proposed adaptation interventions and the RAP participants deliberated and selected the actual interventions for piloting the RAP framework. The selected interventions are Agriculture Information Management System (AIMS) and Sustainable Agriculture Inputs (SAI) (see Methodology for more information).

It should be mentioned here that as the purpose of this paper is to highlight the methodological procedure of the RAP framework, the intent here is to use AIMS and SAI to highlight the procedure, utility and usefulness of the RAP framework in a climate adaptation setting by combining it with broader network analyses. It is not the focus of this paper to assess the effectiveness of the specific adaptation interventions chosen by the actors.

In total 40 participants attended the participatory workshop in Accra (Step 3), with representation from each administrative level: national (45%), region (20%), district (15%) and community (20%) (refer to Figure S2, Supplementary Electronic Material). A team of experienced researchers and workshop facilitators (10), translators (3), note takers (4) and support staff (4), supported the workshop participants.

**Fig. 4:**
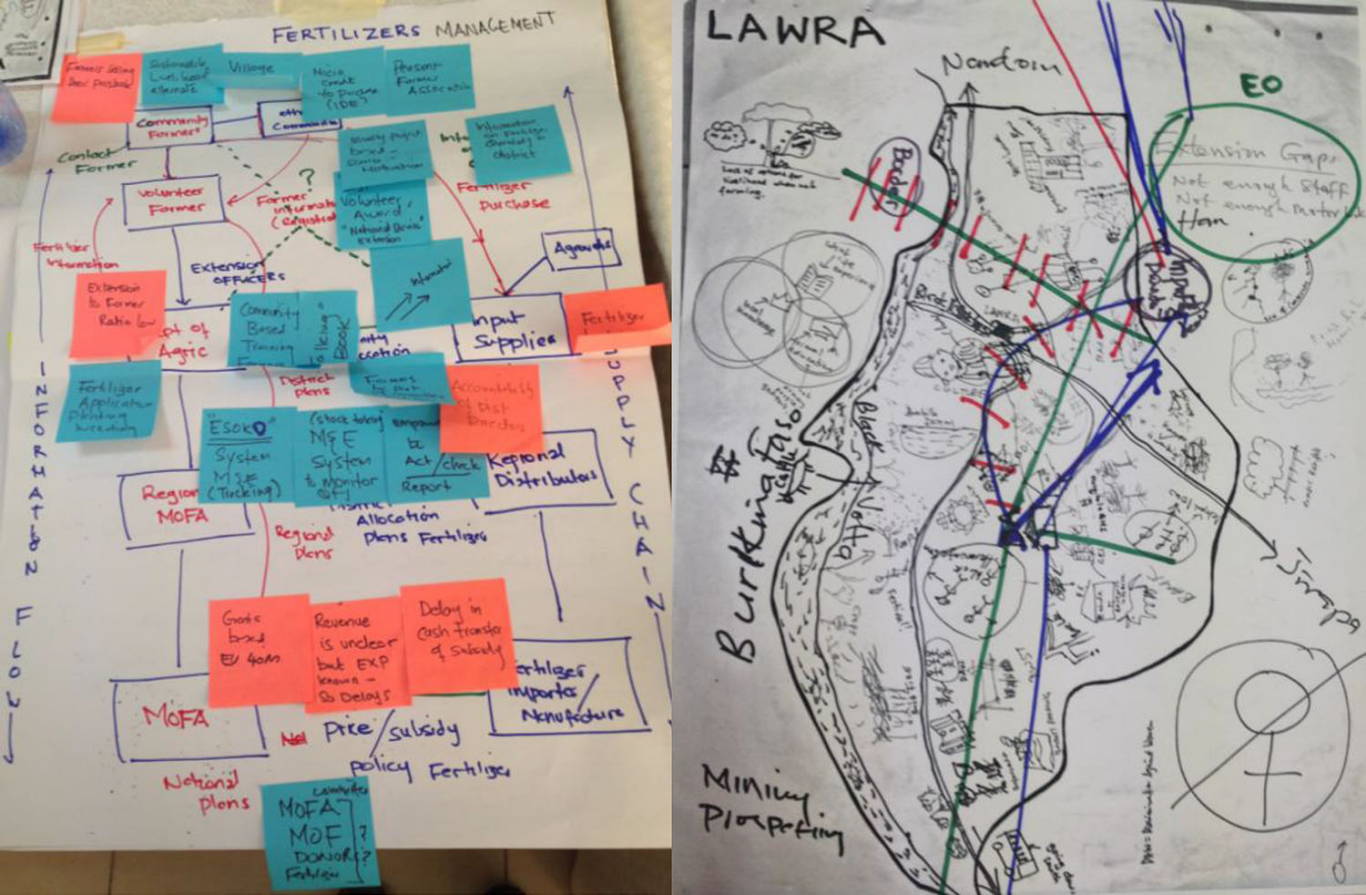
Examples of rich picture of actors and relations collaboratively created by the participants for the selected interventions

The workshop was structured to offer a safe space where actors from different administrative levels and backgrounds could comfortably express their opinions and open up to other perspectives. The participants developed rich pictures of the issues and opportunities at each administrative level within Ghana for AIMS and SAI (see Fig. 4 for examples of the rich pictures created for SAI). Workshop participants collectively identified actors and relations, within and across administrative levels that were considered important for the interventions, and created potential pathways for effective linkage across levels.

**Fig. 5:**
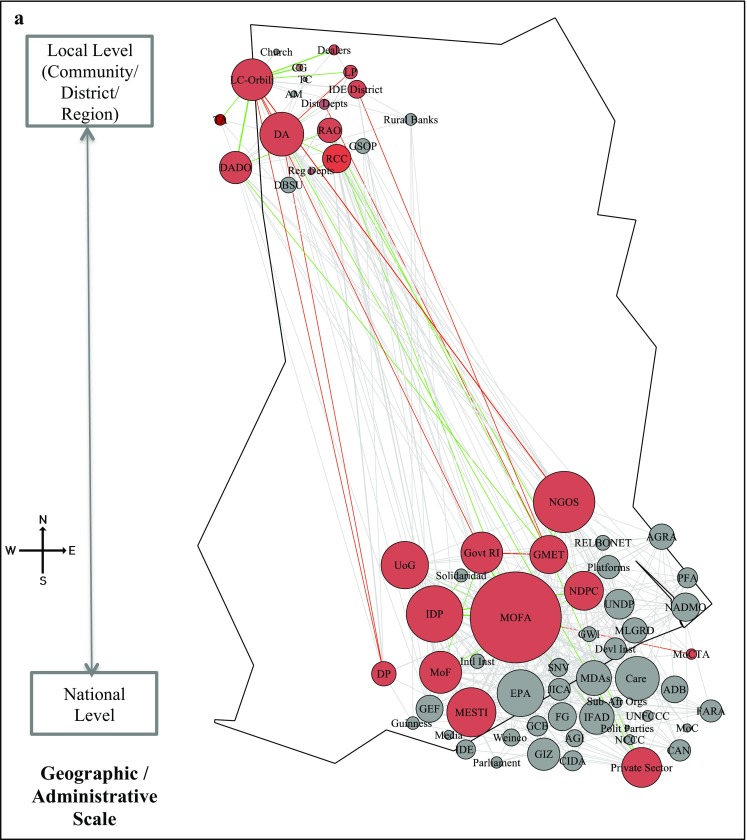

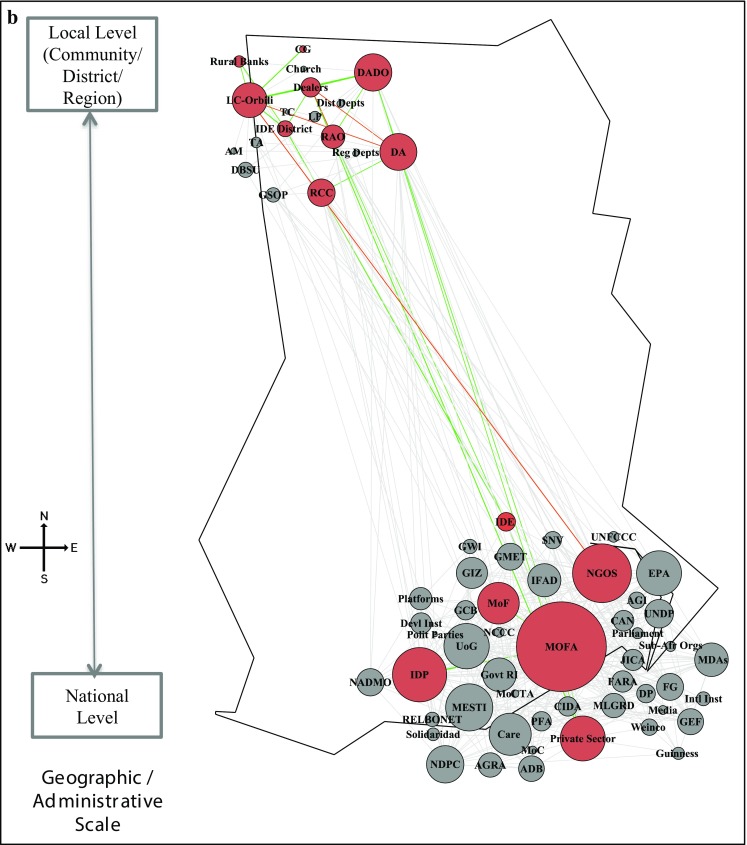
Undirected network map of adaptation actors. **a** Agriculture Information Management System (AIMS). **b** Sustainable Agriculture Inputs (SAI)—Fertilisers management: Nodes on the map represent the 66 actors in the network across levels. The coloured nodes (*red*) represent the actors relevant for the AIMS and SAI interventions. The edges between the actors are weighted to represent multiple relations and strength of interactions between the actors. The *green edges* show the functional links between the actors and the *red edges* represent the missing links that need to be established. The node size of actors is proportional to the number of weighted edges connected to the node. Refer to Table S1 in the Supplementary Electronic Material for names of actors

Step 3 helped create a shared understanding of terms, definitions, actions and the need for a collaborative approach towards planning climate change adaptation interventions, without enforcing any one particular approach. From the 66 actors identified in the baseline mapping (Step 1), workshop participants identified active roles for 24 and 15 actors for AIMS and SAI interventions respectively (highlighted as red coloured nodes in Fig. 5). Participants identified more actors and links for AIMS as technical and traditional knowledge is spread across many actors, whereas under SAI most of the actors/relations are concentrated to the fertiliser supply chain.

Figure 5 and Table 2 show the network maps of AIMS (Fig. 5a, b) and SAI overlaid on the baseline networks and the related network metrics (Step 4). The participatory mapping identified 11 and 5 missing relations (red lines). These missing relations were in addition to existing functional relations of 35 and 24 (green lines), among the 24 and 15 identified actors under AIMS and SAI respectively. The participants did not identify any new actors for both interventions, highlighting that leveraging existing networks is more useful than unnecessarily introducing new actors. Creating only 5% additional relations for AIMS and 4% for SAI of the total weighted relations in the baseline network, a more strongly connected network emerged for the two interventions, also with clearer pathways.

**Table 2:** Network metrics for AIMS and SAI interventions and baseline network

	AIMS	SAI	Baseline network
Size (actors in use)	24	15	66
Existing relations	35	23	350
New relations proposed	11	5	
Overall relations (existing + new)	46	28	
Existing weighted relations	205	159	3054
New weighted relations proposed	158	134	
Overall weighted relations (existing + new)	363	293	
Overall density (existing + new)	0.14	0.22	0.16

We observed stronger density of relations (both for existing and new relations) for SAI at 0.22, compared to 0.14 for AIMS. One plausible explanation for the low density in AIMS is that action and knowledge generated requires the broad participation of many actors from national to local level. Yet not all actors need connections to others for the successful diffusion of knowledge. For example, connecting Ghana Meteorological Agency (GMET) to local NGOs can transfer knowledge to the wider community about local weather patterns and crop selection, rather than tediously communicating directly with each individual. SAI, on the other hand, entails the physical flow of resources (i.e. fertilisers) through the network. As resources are located at one node, these need to flow from one actor to another for the relation to form. This requires direct connections between actors rather than broad links. For example, participants identified missing links between market dealers, community members and official actors (e.g. regional and district departments of agriculture, extension officers) for monitoring fertiliser supplies. This lack of engagement frustrated local farmers that faced fertiliser shortages.

The above experience during Step 3–4 suggest that the RAP framework can point to missing links between actors that can be effectively filled to establish targeted action. While we cannot comment on the effectiveness of these proposed new links, it is reasonable to assume that they improve transparency and effectiveness.

### Developing action plans

In this final step (Step 5), the participants developed action plans for delivering AIMS and SAI. Each action plan identified detailed activities and steps to achieve them, assigned responsibility to actors and setup concrete timelines for undertaking each action, (refer to Table S3–S4 in the Supplementary Electronic Material for more details). The participants suggested ‘champions’ for taking the plans forward, consulting and involving other crucial actors. The action plans included strengthening existing relations and establishing new ones where needed. For example, connecting local communities to procurement officials would improve transparency of action. Although it is difficult to quantify the effectiveness of these relations, they enable pathways within the wider actor network.

For AIMS, the participants debated the challenges within extension services, including knowledge gaps, lack of technology (e.g. localised weather forecasting, crop production and price data dissemination), funding shortages, resource availability and lack of coordination among the various actors. Some even went as far as to say that formal extension services were virtually dead and extension staff had to rely on the help of NGOs to offer such services. Plans for AIMS, therefore, envisioned an improved agricultural extension policy backed by funding to overcome farmers’ current knowledge and support gap. The proposed extension policy would require appropriate contributions from many actors including national ministries, district and regional governments and local communities. It would entail establishing new (and strengthening older) links among all the various actors, especially connecting local communities to central policy-makers in order to reflect local realities in policy design. A pluralistic model was planned to allow service provision by both government and the private sector. This included plans for non-traditional and e-extension services to meet immediate challenges, while maintaining long-term support by extension provision. Regional government actors (e.g. Regional Coordinating Committee and Regional Agriculture Office) were identified as ideal bridges between national and local actors to improve collaboration, transfer knowledge and offer support to farmers across the country. Champions within the local community were identified to receive extension service, adopt new techniques that enhanced traditional practices (e.g. river water pumping systems for dry season farming), and transfer knowledge to other community members. More detailed information about the action plan can be found in Table S3 in the Supplementary Electronic Material.

For SAI, the action plan envisioned setting up a national advocacy group, comprising the key ministries of agriculture and finance. The advocacy group would directly engage with development partners and input suppliers to ensure the timely supply and distribution of fertilisers. The action plan advocated formalising and strengthening existing links among these actors to tackle procurement and supply challenges. Regional and district actors (e.g. Regional Coordinating Committee and District Assembly respectively) were identified as champions for communicating local demand to national actors (e.g. the proposed national advocacy group) and monitoring its subsequent delivery. A key missing link in the fertiliser supply chain identified through RAP was the representation of local farmers on the district’s committee for fertiliser procurement. Farmers blamed the administration for shortages whereas the administration labelled farmers as misusing their allocations by illegally selling subsidised fertiliser for higher margins. By directly involving farmers in the distribution process, the RAP participants envisioned overcoming the mistrust between actors and promoting transparent and fair allocations of inputs (Njoroge et al. 2015). Finally, the SAI action plan emphasised improving the communication between actors by promoting links between disconnected actors and strengthening existing links, rather than just increasing the number of actors (or their relations). More details information about the action plan can be found in Table S4 in the Supplementary Electronic Material.

## Discussion

### Visualising actions across actors and levels

Planning and implementing adaptation is complex because climate change is both a major sustainability challenge and a ‘wicked problem’ that involves many contingent environmental and human variables, playing out across various geographic scales, levels of governance, and sectors of the economy/society.

The low density of connections in Fig. 1 suggests that many potential relations between actors are missing, and as a result important information and resources will not diffuse easily through this network (Bodin and Crona 2009). Actors central to agricultural adaptation can be identified easily, while others that are equally important operating at the periphery of the agricultural sector (e.g. private sector actors) can be harder to identify. Some actors do not even label their activities as related to climate change (e.g. IDE, the central NGO working in Lawra) and could fall outside the regime. Yet many of these peripheral actors could provide valuable contributions through existing knowledge and resource connections.

Deciding which actors to include or exclude, what links to focus on in the design and at what point, is not simple. Visualising this complexity through the rich pictures and network mapping obtained during the RAP process can highlight strengths and contingencies, as well as minimise blind spots and biases. This approach is common to several problem-solving and creative thinking methods because our intuition communicates more easily through impressions and symbols than in words (Bell and Morse 2013). We found that encouraging each of the many actors participating in the RAP process to create pictures helped develop inclusive and robust action plans that are rooted in a manifest social-ecological system of agents, drivers, and processes with existing and potential relations between them. In this way, RAP utilises the collective wisdom of participants to develop feasible and widely acceptable action plans. Visualising adaptation challenges, through rich pictures and logical pathways, helps to break down the climate change adaptation challenge into manageable steps, while offering a structure for developing long-term strategies. Similarly, visualisation helps to identify actors that are important for adaptation but have weak or no links with the adaptation regime. Targeted action plans can be developed to reach out to such actors outside the network in order to acquire new practices and technologies (Kenis and Oerlemans 2008; Powell et al. 1996) that improve adaptation plans.

During the implementation of RAP in Ghana, there were many ‘aha’ moments where participants discovered roles and actors that they were previously unaware of. For example, when illustrating lessons learned from the RAP process one climate NGO official pointed out that “*farmers are undertaking so many activities already in the area of adaptation, which they don’t know is adaptation*” (personal communication 2014). Similarly, participants identified and debated the somewhat ambiguous and overlapping role of regional actors at the local level, which tends to lead to inaction and gaps in offering support. This realization can provide an effective means of supporting existing adaptation processes rather than overriding them with outside models.

Visualising draws attention on existing structures and actors that can be deployed, thus avoiding duplication of structures that is costly and time-consuming. For example, an official from the Environmental Protection Agency (EPA) reinforced the importance of visualisation by pointing out that “*often we underestimate the capacity of the communities. Because [members] know what is happening within their communities, they know how best to adapt. The difficulty is the lack of capacity to be able to see the bigger picture and put it in a more constant framework*” (personal communication 2014). RAP, especially when it incorporates iteratively actors at multiple levels, has the potential to ensure that all key actors in the system do see the big picture, as well as the (potentially changing) positions of other actors’ within the networks over time.

### Creating a shared narrative for adaptation action

Alongside creating the visual network mapping and the participatory planning, a key output of the RAP framework is developing a shared understanding of the challenge and actions required. To reach this understanding explicit consensus is not always needed. Invariably adaptation action takes a policy-driven, forward mapping, top-down approach that starts with policy and assumes “*that policymakers control the organisational, political and technological processes that affect implementation*” (Elmore 1979, p. 603). At the other end, the bottom-up and backward mapping approaches acknowledge that while central programmes offer more opportunities to local actors, they are only one of the considerations in the implementation process (Klijn 2008).

As a result when designing/implementing adaptation actions it is not simply about deciding whether to take either a top-down or a bottom-up vision, but also to recognise that responses lie across levels, especially in strategic collaboration and coordination or roles and responsibilities. An official from the Ministry of Finance (MoF) reinforced this multilevel approach by saying that “*we don’t do top*-*down planning. [Instead] it is top*-*down and bottom*-*up, because the policy comes from the top, but the activities come from the ground to satisfy those policies*” (personal communication 2014). However, this understanding is also simplistic as the implementation of policy is often spread across actors and levels, rather than confined to just a few actors at a particular level.

However, most of the participants had little faith in the government’s ability to implement adaptation interventions alone and stressed that a shared approach is needed. As the head of a farmer interest group put it: “*all the policies are actually good, it is the implementation that is problematic…We have realized that it’s very difficult for a lot of policies to reach the grass roots farmer*” (personal communication 2014). The official from the office of the National Development Planning Commission reiterated that the “*main problem in Ghana is not the lack of ideas, but their implementation and coordination for effective, efficient and smooth implementation*” (personal communication 2014). Hence it is important to include others, “*if you are going into work with a community, you need to involve them, otherwise it will not be sustainable*”, as an academic from the University of Ghana stated (personal communication 2014). Thus, through the RAP process a shared narrative of ongoing involvement of the community and other actors was recognised as a prerequisite for sustaining adaptation processes.

Having actors from all levels sitting across the same table breaks barriers, and helps frame better solutions in terms of actions and roles. This allows for an inclusive and multidimensional approach for the design of adaptation interventions based on shared knowledge. For AIM, the multidimensional approach entailed the improved collection of baseline data on agriculture extension support to inform and update the current policy. Creating such a ‘deliberative space’ (Hendriks 2002) is important for actors to dissect successfully the issues at stake.

The RAP approach builds on the premise that participants are most knowledgeable about their own levels/actions, and it is by respecting and incorporating their diverse views that a shared approach evolves. Facilitating participants to take an emphatic view of the worldviews of actors from different levels within the planning structures can help move the discussion away from the traditional hierarchy of power (i.e. from higher policy level to lower community level) to one of shared responsibility and mutual dependence. During the RAP process, the officials from the Regional Coordinating Committee that are empowered with oversight of districts acknowledged the challenges of enforcing direct authority on the districts, as well as their lack of response. A more prudent approach to engage districts would be for the region to act as facilitator of knowledge sharing across districts, rather as an enforcer of central policy.

It then becomes possible to capture the interests and understanding of different actors about the issue without enforcing a single definition (i.e. multi-vocality), where many meanings can be drawn from the same process (Ferraro et al. 2015). The interpretation of adaptation and of the perceived roles of actors often varies along national to local planning structures. In the RAP process, national actors identified adaptation action for AIMS in terms of extension policy decisions, but at the local level many of the production choices were influenced by the availability of official extension support or the presence of NGOs. Having actors sit across the same table helped develop a broad consensus for action without enforcing a single definition of adaptation, thus respecting each other’s view point. Where one level stops, participants from other levels step into take adaptation planning to a logical conclusion. For example, the traditional chief of Orbili highlighted the need for multiple actors by saying that “*the ordinary farmer knows that something is happening. They don’t know what to do with [that knowledge]*” (personal communication 2014).

This shared approach is the foundation of the RAP framework and helps participants overcome translation problems and the knowledge-action gaps. For example, while doing fieldwork at Lawra we observed that other environmental issues such as sanitation are often incorrectly framed as climate adaptation. The RAP stresses the continuity of the process based on shared knowledge rather than deferring action or incorrectly representing others interests. This moves the discussion away from blame, to one of empathy and collective purpose. This multi-actor discussion acknowledges the inherent challenges and limitations at each level, thus allowing the RAP participants to move collaboratively towards solutions/adaptation interventions that satisfy everyone. The solutions may not always follow unified definition but are acceptable if these meet the needs of the participants across levels for adaptation. This also gives the underrepresented and marginal actors a voice. For example, many government officials were for the first time collaborating directly with farmers, and so had the opportunity to explain their positions and appreciate local challenges.

### Leading and sharing responsibility for adaptation action

A problem in developing a shared understanding of the adaptation challenge (and the responses required) is deciding *who* leads the process and *how* responsibilities are shared among the various actors and participants. During the RAP process, identifying the most appropriate actors to champion the activities was difficult, as their roles and scopes for action are not always clear. Conventional organisations such as private sector for-profit organisations usually have a definite line of authority where action, activities, and roles are clear (Daft et al. 2010), while adaptation regimes need to be dynamic and fluid (Folke et al. 2005).

Here the role of boundary and bridging actors (Berkes 2009; Cash and Moser 2000; Granovetter 1973), who can work across levels, is important to support both the other actors and the adaptation intervention itself. Bridging actors, because of their position, help translate ideas about adaptation as the emphasis changes from one level to another (e.g. from the national to the local level). According to Cash et al. (2006), effective systems perform many functions that contribute significantly to boundary management (e.g. knowledge co-production, mediation, translation, and negotiation). Effective mediation appears to be the most important element in ensuring legitimacy through increased transparency, by bringing all perspectives to the table, providing rules of conduct, and establishing criteria for decision making (Ostrom 2005). Regional governance in Ghana, for example, has the role of coordinating and translating into local action the information offered from the national government, and yet remains underutilised due to the lack of formal mandate, power or influence. One regional planning officer expressed serious reservations and frustration about the current fragmented approach, despite existing planning structures. As he stated “…*if you bypass the region and go directly to the district, chances are that you will have difficulties with them. They may not see, or may not have the interest that you wish them to get*” (personal communication 2014).

Boundary organisations also support adaptation processes by maintaining the momentum created through the RAP, but can slow down when actors at any level fail to follow through. Clarifying these roles and keeping an element of accountability, feedback and flexibility, maintains effective action. However, this is challenging as was articulated by a regional NGO. Specifically “*many of these planning officers [at the district] do not have the requisite knowledge in participatory methodologies. This makes it difficult for them to engage people and collect information for planning*” (personal communication 2014). Our research demonstrates that by understanding these channels, roles, links and the actions taken, the adaptation regime is made more robust, resilient, responsive, and functional. Identifying champions and allocating responsibility moves the process from theory to formal and concrete responses.

### Practical considerations for planning RAP applications

The RAP framework facilitates the participatory development of shared action plans and tests their robustness against local realities. The plans are built based on empirical data, and supported by input from people across multiple administrative levels. However as any participatory process the RAP framework can require significant resources (in terms of time, budget and capacity) for its effective implementation.

Essential to RAP is a knowledgeable project leader who is sensitive to the challenges and appreciates the social, economic, political and cultural differences among the participants. While it is hard to quantify the time, resources and human commitment to implement RAP as they can vary depending upon the nature and context of the research setting, they can be substantial. In our case, RAP application required a few months of primary data collection to identify actor relations and local challenges, planning workshops (including selecting and inviting participants), developing adaptation interventions with expert input for RAP applications and securing the financial commitment of hosting community and RAP workshops (refer to Table 1).

However in other settings the required resources might be much less. For instance, where strong baseline data is already available, the time required can be reduced substantially. Similarly, RAP workshop format may be altered to focus on one region or theme in more depth. In short, RAP offers a structured process but one that matures with time, and resources through repeated application.

## Conclusions

The RAP framework piloted in this paper offers a practical methodology for developing and implementing sound plans to deliver climate adaptation in agricultural settings. RAP is a highly participatory process that brings together stakeholders from different sectors and administrative levels to identify adaptation pathways and disconnections within the existing networks of actor. This enables them to identify deficits, opportunities and actions to improve the coordination (and ultimately the conditions) under which vulnerable populations cope with climate change.

The RAP framework highlights three important elements: (1) visualise actions across levels and actors to reduce the complexity of adaptation responses; (2) offer a shared space to actors from different levels to think and create collective narratives for adaptation without demanding explicit consensus; (3) identify key actors to allocate roles and responsibility for delivering effective adaptation plans through a collaborative process. By the end of the RAP process, participants should be clear on what actions are needed and who has responsibility for each.

Our results from piloting the RAP framework in the context of agriculture in Ghana demonstrate that the responsibility to plan and implement adaptation actions generally lies beyond the remit of a single actor (e.g., the national government). In some cases coordinating action is complex, requiring concerted efforts by actors to establish formal channels of communication through Ghana’s formal administrative structures. While in other cases it is simply bringing together disconnected actors (across and between the national to the local level in Ghana) to take action. Using existing networks and actors engenders the necessary collaborative understanding and vision to motivate collective action that promotes unity, coherence, empowerment, efficiency and effectiveness in climate action. By adopting a participatory approach across all levels, a shared understanding of adaptation is developed, with strong ownership of the process as witnessed in the robust adaptation plans developed for AIMS and SAI by the participants of the RAP workshop.

Our application of RAP also demonstrates that robust action is essential to address the complexities of climate change by balancing the need for short-term responses and adapting to the long-term consequences. The RAP framework thus offers a valuable tool to policy makers, planners and other stakeholders to develop robust plans that involve many actors spread across multiple levels. However, as the RAP framework is new and evolving, more applications (i.e. case studies) are needed in different settings, sectors, and geographic contexts to evaluate fully its broader utility. Given the promising performance of the framework as reported in this paper it seems to be worth more attention.

## Electronic supplementary material

Below is the link to the electronic supplementary material.

Supplementary material 1 (DOCX 2680 kb)

## Footnotes

1 Agriculture is the mainstay of Ghana’s economy (a lower middle-income country), occupying over half of its population, mostly as smallholder farmers (Government of Ghana 2010).

2 Length represents how long actors have been interacting. Frequency captures how often actors interact (e.g. monthly, quarterly or yearly). Type highlights the different relationship between the actors (i.e. resource, knowledge, services, and connections.) Usefulness determines the strength of relationships between actors, as scored by the actors.

## References

[CR1] Adger WN, Huq S, Brown K, Conway D, Hulme M (2003). Adaptation to climate change in the developing world. Prog Dev Stud.

[CR2] Adger WN, Arnell WN, Tompkins LE (2005). Successful adaptation to climate change across scales. Glob Environ Change.

[CR3] Adner R (2006). Match your innovation strategy to your innovation ecosystem. Har Bus Rev.

[CR4] Antwi-Agyei P, Fraser EDG, Dougill AJ, Stringer LC, Simelton E (2012). Mapping the vulnerability of crop production to drought in Ghana using rainfall, yield and socioeconomic data. Appl Geogr.

[CR5] Bell S, Morse S (2013). How people use rich pictures to help them think and act. Syst Pract Action Res.

[CR6] Berkes F (2009). Evolution of co-management: role of knowledge generation, bridging organizations and social learning. J Environ Manage.

[CR7] Biernacki P, Waldorf D (1981). Snowball sampling: problems and techniques of chain referral sampling. Sociol Methods Res.

[CR8] Bodin Ö, Crona BI (2009). The role of social networks in natural resource governance: what relational patterns make a difference?. Glob Environ Change.

[CR9] Bodin Ö, Crona B, Ernstson H (2006). Social networks in natural resource management: what is there to learn from a structural perspective. Ecol Soc.

[CR10] Bolman LG, Deal TE (2013). Reframing organizations: artistry, choice and leadership.

[CR11] Borgatti SP, Mehra A, Brass DJ, Labianca G (2009). Network analysis in the social sciences. Science.

[CR12] Brown T (2009). Change by design.

[CR13] Bryson JM (2004). What to do when stakeholders matter: stakeholder identification and analysis techniques. Public Manag Rev.

[CR14] Burt RS (2002). Structural holes: the social structure of competition.

[CR15] Cash DW, Moser SC (2000). Linking global and local scales: designing dynamic assessment and management processes. Glob Environ Change.

[CR16] Cash D, Adger N, Berkes F, Garden P, Lebel L, Olsson P, Pritchard L, Young O (2006). Scale and cross-scale dynamics: governance and information in a multilevel world. Ecol Soc.

[CR17] Cassidy L, Barnes GD (2012). Understanding household connectivity and resilience in marginal rural communities through social network analysis in the village of Habu, Botswana. Ecol Soc.

[CR18] Chambers R (1992) Rural appraisal: rapid, relaxed and participatory. Discussion Paper 311. Institute of Development Studies, Brighton

[CR19] Chaudhury AS, Helfgott A, Thornton TF, Sova C (2016). Participatory adaptation planning and costing: applications in agricultural adaptation in western Kenya. Mitig Adapt Strat Glob Change.

[CR20] Chaudhury AS, Ventresca MJ, Thornton TF, Helfgott A, Sova C, Baral P, Rasheed T, Ligthart J (2016). Emerging meta-organisations and adaptation to global climate change: evidence from implementing adaptation in Nepal, Pakistan and Ghana. Glob Environ Change.

[CR21] Chaudhury AS, Thornton TF, Helfgott A, Ventresca MJ, Sova C (2017). Ties that bind: local networks, communities and adaptive capacity in rural Ghana. J Rural Stud.

[CR22] Cooke B, Kothari U (2001). Participation: the new tyranny?.

[CR23] Daft RL, Murphy J, Willmott H (2010). Organization theory and design.

[CR24] Deressa TT, Hassan RM, Ringler C, Alemu T, Yesuf M (2009). Determinants of farmers’ choice of adaptation methods to climate change in the Nile Basin of Ethiopia. Glob Environ Change.

[CR25] Elmore RF (1979). Backward mapping: implementation research and policy decisions. Politi Sci Q.

[CR26] Etwire PM, Al-Hassan RM, Kuwornu JK, Osei-Owusu Y (2013). Application of livelihood vulnerability index in assessing vulnerability to climate change and variability in Northern Ghana. J Environ Earth Sci.

[CR27] Etzion D, Gehman J, Ferraro F, Avidan M (2015). Unleashing sustainability transformations through robust action. J Clean Prod.

[CR28] FAO (2012). The state of food and agriculture—investing in agriculture for a better future.

[CR29] Ferraro F, Etzion D, Gehman J (2015). Tackling grand challenges pragmatically: robust action revisited. Organ Stud.

[CR30] Folke C, Carpenter S, Elmqvist T, Gunderson L, Holling CS, Walker B (2002). Resilience and sustainable development: building adaptive capacity in a world of transformations. AMBIO. J Human Environ.

[CR31] Folke C, Hahn T, Olsson P, Norberg J (2005). Adaptive governance of social-ecological systems. Annu Rev Environ Resour.

[CR32] Füssel HM (2007). Adaptation planning for climate change: concepts, assessment approaches, and key lessons. Sustain Sci.

[CR33] Gibson CC, Ostrom E, Ahn TK (2000). The concept of scale and the human dimensions of global change: a survey. Ecol Econ.

[CR34] Goodman LA (1961). Snowball sampling. Inst Math Stat.

[CR35] GovernmenT of Ghana (2010) Medium term agriculture investment plan (METASIP) 2011–2015. http://www.mofa.gov.gh/site/?page_id=2754. In: Ministry of Food and Agriculture (ed) Ghana

[CR36] Government of Ghana (2015) Ghana’s third national communication to the UNFCCC, 2015. http://www.unfccc.int/resource/docs/natc/ghanc3.pdf. In: Agency EP (ed) Ghana

[CR37] Granovetter MS (1973). The strength of weak ties. Am J Sociol.

[CR38] Green M, Kothari U, Minogue M (2002). Development theory and practice. Social development: issues and approaches: critical perspectives.

[CR39] Hajer MA, Wagenaar H (2003). Deliberative policy analysis: understanding governance in the network society.

[CR40] Hanneman RA, Riddle M (2005) Introduction to social network methods. http://faculty.ucr.edu/~hanneman/. University of California Riverside, Riverside

[CR41] Hanneman RA, Riddle M (2011). A brief introduction to analyzing social network data. SAGE Handb Soc Netw Analy.

[CR42] Helfgott A, Sova C, Thorn J, Chaudhury A, Bailey M, Vervoort J, Ademiluyi A, Grift EV (2014). Multi-level integrated adaptation governance Ghana workshop report.

[CR43] Hendriks C (2002). Institutions of deliberative democratic processes and interest groups: roles, tensions and incentives. Aust J Public Adm.

[CR44] Hill M, Engle NL (2013). Adaptive capacity: tensions across scales. Environ Policy Gov.

[CR45] Ibarra H, Andrews SB (1993). Power, social influence, and sense making: effects of network centrality and proximity on employee perceptions. Adm Sci Q.

[CR46] IPCC (2014) Africa. In: Climate Change 2014: Impacts, adaptation, and vulnerability. Part B: regional aspects. contribution of working group II to the fifth assessment report of the intergovernmental panel on climate change In: Field CB, VR Barros DJ Dokken KJ Mach MD Mastrandrea TE Bilir M Chatterjee KL Ebi YO Estrada RC Genova B Girma ES Kissel AN Levy S Maccracken PR Mastrandrea, LL White (ed) http://www.ipcc.ch/pdf/assessment-report/ar5/wg2/WGIIAR5-Chap22_FINAL.pdf. IPCC, Cambridge

[CR47] Kenis P, Oerlemans L, Cropper S, Ebers M, Huxham C, Ring PS (2008). The social network perspective: understanding the structure of cooperation. Oxford handbook of inter-organizational relationships.

[CR48] Klijn E-H, Cropper S, Ebers M, Huxham C, Ring PS (2008). Policy and implementation networks: managing complex interactions. The Oxford handbook of inter—organizational relations.

[CR49] Lazarus RJ (2008). Super wicked problems and climate change: restraining the present to liberate the future. Cornell L Rev.

[CR50] Lempert RJ, Schlesinger ME (2000). Robust strategies for abating climate change. Clim Change.

[CR51] Lewis P (1992). Rich picture building in the soft systems methodology. Eur J Inform Syst.

[CR52] Lin BB (2011). Resilience in agriculture through crop diversification: adaptive management for environmental change. Bioscience.

[CR53] Marin A, Wellman B, Scott J, Carrington PJ (2011). Social network analysis: an introduction. The SAGE handbook of social network analysis.

[CR54] Marsden PV (1990). Network data and measurement. Ann Rev Sociol.

[CR55] McCarty C, Bernard HR (2003). Social network analysis. Encyclopedia of community: from the village to the virtual world.

[CR56] Mcgee R, Kothari U, Minogue M (2002). Participating in development. Development theory and practice: critical perspectives.

[CR57] Mcsweeney C, New M, Lizcano G (2010) UNDP Climate change country profiles, Ghana http://www.geog.ox.ac.uk/research/climate/projects/undp-cp/. Accessed Jan 10 2017

[CR58] Mermet L (2011) Strategic environmental management analysis: addressing the blind spots of collaborative approaches. Working Paper No 5. IDDRI

[CR59] Mikkelsen B (2005). Methods for development work and research: a new guide for practitioners, New Delhi.

[CR60] Monk A, Howard S (1998). Methods and tools: the rich picture: a tool for reasoning about work context. Interactions.

[CR61] Mosse D (2004). Is good policy unimplementable? Reflections on the ethnography of aid policy and practice. Dev Change.

[CR62] Naab JB Sissoko K Zougmore R, Traore B, Amadou M, Moussa AS, Forch W, Garlick C, Ochieng S Kristjanson P, Thornton PK (2011) Summary of baseline household survey results: Lawra-Jirapa, Ghana—http://ccafs.cgiar.org/publications/summary-baseline-household-survey-results-lawra-jirapa-ghana. VIiS3b54x_Y. Copenhagen, Denmark: CGIAR Research Program on Climate Change, Agriculture and Food Security (CCAFS)

[CR63] Nair S, Howlett M (2016). From robustness to resilience: avoiding policy traps in the long term. Sustain Sci.

[CR102] Nelson GC, Rosegrant MW, Koo J, Robertson R, Sulser T, Zhu T, Ringler C, Msangi S, Palazzo A, Batka M (2009) Climate change: impact on agriculture and costs of adaptation. Washington DC: The international food policy research institute. http://www.ifpri.org/sites/default/files/publications/pr21.pdf

[CR64] Newman L, Dale A (2005). Network structure, diversity, and proactive resilience building: a response to Tompkins and Adger. Ecol Soc.

[CR65] Njoroge R, Birech R, Arusey C, Korir M, Mutisya C, Scholz RW (2015). Transdisciplinary processes of developing, applying, and evaluating a method for improving smallholder farmers’ access to (phosphorus) fertilizers: the SMAP method. Sustain Sci.

[CR66] Ostrom E (2005). Understanding institutional diversity.

[CR67] Ostrom E (2010). A multi-scale approach to coping with climate change and other collective action problems. Solutions.

[CR68] Ostrom E (2010). Polycentric systems for coping with collective action and global environmental change. Glob Environ Change.

[CR69] Padgett JF, Ansell CK (1993). Robust action and the rise of the Medici, 1400–1434. Am J Sociol.

[CR70] Padgett JF, Powell WW (2012). The emergence of organizations and markets.

[CR71] Powell WW, Koput KW, Smith-Doerr L (1996). Interorganizational collaboration and the locus of innovation: networks of learning in biotechnology. Adm Sci Q.

[CR72] Reid H, Huq S (2007) Community-based adaptation: a vital approach to the threat climate change poses to the poor. International Institute for Environment and Development (IIED). Briefing Paper. IIED, London

[CR73] Sandström AC, Rova CV (2009). The network structure of adaptive governance—a single case study of a fish management area. Int J Commons.

[CR74] Schipper ELF, Ayers J, Reid H, Huq S, Rahman A (2014). Community-based adaptation to climate change: scaling it up.

[CR75] Schlenker W, Lobell DB (2010). Robust negative impacts of climate change on African agriculture. Environ Res Lett.

[CR76] Scott JC (1998). Seeing like a state: how certain schemes to improve the human condition have failed.

[CR77] Scott J, Carrington PJ (2011). The SAGE handbook of social network analysis.

[CR78] Silverman D (2013). Doing qualitative research: A practical handbook.

[CR79] Smit B, Burton I, Klein RT, Wandel J (2000). An anatomy of adaptation to climate change and variability. Clim Change.

[CR80] Sova CA, Helfgott A, Chaudhury AS, Matthews D, Thornton TF, Vermeulen SJ (2015). Multi-level stakeholder influence mapping: visualizing power relations across actor levels in Nepal’s agricultural climate change adaptation regime. Syst Pract Action Res.

[CR81] Sova CA, Thornton TF, Zougmore R, Helfgott A, Chaudhury AS (2016). Power and influence mapping in Ghana’s agricultural adaptation policy regime. Clim Dev.

[CR82] Stanturf J, Warren M, Charnley Jr S, Polasky SC, Goodrick SL, Armah F, Nyako YA (2011). Ghana climate change vulnerability and adaptation assessment.

[CR83] Stein C, Ernstson H, Barron J (2011). A social network approach to analyzing water governance: the case of the Mkindo catchment, Tanzania. Physics Chem Earth Parts A/B/C.

[CR84] Tambo JA, Abdoulaye T (2012). Climate change and agricultural technology adoption: the case of drought tolerant maize in rural Nigeria. Mitig Adapt Strat Glob Change.

[CR85] Vervoort JM, Thornton PK, Kristjanson P, Förch W, Ericksen PJ, Kok K, Ingram JSI, Herrero M, Palazzo A, Helfgott AES, Wilkinson A, Havlík P, Mason-D’croz D, Jost C (2014). Challenges to scenario-guided adaptive action on food security under climate change. Glob Environ Change.

[CR86] Vignola R, McDaniels TL, Scholz RW (2013). Governance structures for ecosystem-based adaptation: using policy-network analysis to identify key organizations for bridging information across scales and policy areas. Environ Sci Policy.

[CR87] Wasserman S (1994). Social network analysis: methods and applications.

[CR88] Wellman B, Wortley S (1990). Different strokes from different folks: community ties and social support. Am J Sociol.

[CR89] Wittmayer JM, Schäpke N (2014). Action, research and participation: roles of researchers in sustainability transitions. Sustain Sci.

