# Supplementary material for: Applying the robust adaptation planning (RAP) framework to Ghana’s agricultural climate change adaptation regime
Source: Sustain Sci. 2017 Aug 19;12(5):657–76. doi: 10.1007/s11625-017-0462-0 (PMC6086261; doi:10.1007/s11625-017-0462-0)
Supplement: Supplementary file 1 — Supplementary material 1 (DOCX 2680 kb) [file 11625_2017_462_MOESM1_ESM.docx]

**SUPPLEMENTARY ELECTRONIC MATERIAL**

**ACTORS IN BASELINE NETWORK (IN ALPHABETIC ORDER)**

**Table S1:**

| **Label** | **Name** | **Category** | **Weighted Degree** |
| --- | --- | --- | --- |
| ADB | Agriculture Development Bank | Private Sector | 54 |
| AGI | Association of Ghana Industry | Associations/Media | 32 |
| AGRA | Alliance for a Green Revolution in Africa | International Institutions | 51 |
| AM | Assembly Members (Lawra) | District Government | 7 |
| CAN | CAN Ghana | NGOs | 42 |
| Care | Care International | NGOs | 93 |
| CG | Community Groups | Community/Traditional Auth | 9 |
| Church | Church | Community/Traditional Auth | 6 |
| CIDA | Canadian International Development Agency | International Institutions | 39 |
| DA | District Assembly (Lawra) | District Government | 78 |
| DADO | District Agriculture Department Office | District Government | 66 |
| DBSU | District Business Support Unit | District Government | 29 |
| Dealers | Market Dealers | Private Sector | 16 |
| Devl Inst | The Development Institute | NGOs | 43 |
| Dist Depts | District Departments | District Government | 10 |
| DP | Development Programmes-SADA/NRGP | Programmes/Projects | 37 |
| EPA | Environmental Protection Agency | National Government | 100 |
| FARA | Forum of Agricultural Research in Africa | International Institutions | 44 |
| FG | Farmer Groups | Associations/Media | 57 |
| GCB | Ghana Cocoa Board | National Government | 39 |
| GEF | Global Environmental Facility | International Institutions | 55 |
| GIZ | GIZ | NGOs | 67 |
| GMET | Ghana Meteorological Agency | National Government | 55 |
| Govt RI | Government Research Institutes | Academia / Research | 67 |
| GSOP | Ghana Social Opportunity Project | Programmes/Projects | 27 |
| Guinness | Guinness Ghana | Private Sector | 22 |
| GWI | Ghana Wildlife Society | NGOs | 27 |
| IDE | IDE Ghana | NGOs | 37 |
| IDE District | IDE District Office | NGOs | 31 |
| IDP | International Development Partners | International Institutions | 113 |
| IFAD | International Fund for Agriculture Development | International Institutions | 72 |
| Intl Inst | International Institutions (Others) | International Institutions | 35 |
| JICA | Japan International Cooperation Agency | International Institutions | 43 |
| LC-Orbili | Local Community -Orbili | Community/Traditional Auth | 60 |
| LP | Local Projects | Programmes/Projects | 17 |
| MDAs | Government Ministries, Depts and Agencies | National Government | 73 |
| Media | Media | Associations/Media | 14 |
| MESTI | Ministry of Environment, Sci, Tech & Innov | National Government | 105 |
| MLGRD | Ministry of Local Govt and Rural Development | National Government | 51 |
| MoC | Ministry of Communication | National Government | 12 |
| MoCTA | Ministry of Chieftaincy and Traditional Affairs | National Government | 11 |
| MoF | Ministry of Finance | National Government | 89 |
| MOFA | Ministry of Food and Agriculture | National Government | 189 |
| NADMO | National Disaster Management Organization | National Government | 58 |
| NCCC | National Climate Change Committee | National Government | 16 |
| NDPC | National Development Planning Commission | National Government | 73 |
| NGOS | Non-Governmental Organizations (Others) | NGOs | 123 |
| Parliament | National Parliament | National Government | 19 |
| PFA | Peasant Farmers Association | NGOs | 39 |
| Platforms | Climate Platforms | Associations/Media | 46 |
| Polit Parties | Political Parties | Associations Media | 3 |
| Private Sector | Private Sector | Private Sector | 84 |
| RAO | Regional Agriculture Office | Regional Government | 41 |
| RCC | Regional Coordinating Council | Regional Government | 58 |
| Reg Depts | Regional Departments | Regional Government | 7 |
| RELBONET | Religious Board Network | NGOs | 27 |
| Rural Banks | Rural Banks | Private Sector | 20 |
| SNV | SNV | NGOs | 34 |
| Solidaridad | Solidaridad | NGOs | 23 |
| Sub-Afr Orgs | Sub-African Organisations | International Institutions | 17 |
| TA | Traditional Authority | Community/Traditional Auth | 18 |
| TC | Town Committees | District Government | 3 |
| UNDP | United Nations Development Programme | International Institutions | 61 |
| UNFCCC | International Climate Agencies - UNFCCC | International Institutions | 18 |
| UoG | University of Ghana | Academia/Research | 110 |
| Weinco | Weinco - Private Sector | Private Sector | 32 |
|  |  | **Total** | **3,054** |

**LIST OF PARTICIPANTS FOR PARTICIPATORY WORKSHOP –ACCRA**

**Table S2:**

|  | **National Level Participants** |  |
| --- | --- | --- |
| 1 | Ministry of Food and Agriculture | Government |
| 2 | Ministry of Food and Agriculture | Government |
| 3 | The Development Institute | NGO |
| 4 | National Development Planning Commission | Government |
| 5 | National Development Planning Commission | Government |
| 6 | Peasant Farmers Association | NGO |
| 7 | Ghana Meteorological Agency | Government |
| 8 | African Development Bank | Private Sector |
| 9 | FAO Ghana | International Institution |
| 10 | FAO Ghana | International Institution |
| 11 | Ministry of Finance | Government |
| 12 | Engineers without Borders | NGO |
| 13 | RELBONET | NGO |
| 14 | WWF | NGO |
| 15 | The International Stingless Bee Centre | NGO |
| 16 | CSIR-ARI | Academia / Research Institute |
| 17 | CSIR-ARI | Academia / Research Institute |
| 18 | CSIR-ARI | Academia / Research Institute |
|  |  |  |
|  | **Regional Level Participants** |  |
| 19 | CSIR-ARI | Academia / Research Institute |
| 20 | CARE | NGO |
| 21 | CIDA | International Institution |
| 22 | CSIR-SARI | Academia / Research Institute |
| 23 | CSIR-ARI | Academia / Research Institute |
| 24 | CARE | NGO |
| 25 | RCC Planning Officer | Government |
| 26 | ACEP | NGO |
|  |  |  |
|  | **District Level Participants** |  |
| 27 | District Agriculture Department | Government |
| 28 | District Agriculture Department | Government |
| 29 | Lawra District Assembly | Government |
| 30 | Lawra District Assembly | Government |
| 31 | IDE | NGO |
| 32 | CSIR-ARI | Academia / Research Institute |
|  |  |  |
|  | **Community Level Participants** |  |
| 33 | Orbili | Community |
| 34 | Orbili | Community |
| 35 | Orbili | Community |
| 36 | Orbili | Community |
| 37 | Teater Farmers Coop | Community |
| 38 | Boo Nayor Disabled Farmers Association | Community |
| 39 | NANDIREP | NGO |
| 40 | UDS Institute of Continuing Education | Academia / Research Institute |

**Figure S1:**

**Figure S2:**

The workshop was supported by a team of experienced researchers and workshop facilitators (10), translators (3), note takers (4) and support staff (4).

**VISUAL AGENDA FOR GHANA MULTI-LEVEL INTEGRATED ADAPTATION GOVERNANCE WORKSHOP**

**Figure S3:**

Figure S3 shows the visual agenda of the workshop spread over three days. However, the structure and duration can be varied depending on the individual circumstances.

**PROPOSED ACTION PLAN FOR AGRICULTURE INFORMATION SYSTEM (AIMS)**

**Table S3:**

| **Action** | **Champion** | **Approval** | **Consulted** | **Informed** | **Time** |
| --- | --- | --- | --- | --- | --- |
| Advocacy of Extension Gap | Civil Society, Peasant Farmers Association, Development Institute (DI) | Ministry of Food and Agriculture (MOFA) and Policy Planning Division (PPMED) | AGRA NGO (Have done similar advocacy in this area) | National Development Planning Commission (NDPC) |  |
| Improving investment in Agriculture  (10% allocation of budget under Maputo Declaration on Agriculture) | AGRA (have done work in this area) |  | AGRA |  |  |
| Leadership for agriculture extension policy development | MOFA – Extension Service Directorate and PPMED | Chief Director of MOFA | PPMED | Minister of Agriculture for buy-in |  |
| Document Review (FASDEP II, METASIP, 2002 Extension Policy) | PPMED Policy Unit | Director PPMED |  |  |  |
| Buy-in and consultation from the Agriculture Sector Working Group | Relevant NGO |  | Agricultural Sector Annual Review | PPMED Head of Policy |  |
| Consulting/ modifying existing curriculum | National Accreditation Board, Ghana Education Service Tertiary Education Unit |  | University of Cape Coast, Ghana Legon, UDS | CSIR Graduate School (forthcoming) |  |
| Baseline data on existing extension services | Ghana Statistical Services, MoFA Statistical Research Department (SRID) |  | SRID, Regional M&E officers, and District MIS officers |  |  |
| Use of E-extension tools like Esoko | MOFA E-agriculture initiative via the IT department and Chief Director’s Office |  |  |  |  |
| Improved national service participation in extension and on-the-job training | District Director of Agriculture and the National Service Secretariat |  | District Assembly |  |  |
| Improved frequency of regional meetings | MOFA Regional Director of Agriculture, CSIR Coordinator in Upper West |  |  |  |  |
| Increased farmer field schools and demonstrations | Project Driven [Development partners], District Department of Agriculture, CSIR |  | NGOs, and CSOs, FAO |  |  |
| Improved community organization for adoption | Community Contact, Extension Supervisor |  | Chief, Sub-chiefs, and District units |  |  |

**PROPOSED ACTION PLAN FOR SUSTAINABLE AGRICULTURE INPUTS (SAI)**

**Table S4:**

| **Action** | **Champion** | **Approval** | **Consulted** | **Informed** | **Time** |
| --- | --- | --- | --- | --- | --- |
| **Delay in cash transfer of subsidy by government to importers/manufactures, leading to delay in fertiliser availability**  (*The reason is that while expenditure is clear, the revenue is an estimate that often falls short. Part is the dependence on foreign grants to plug budget deficit (example Euro 40m held by EU))* | | | | | |
| Setup of advocacy group comprising ministries of agriculture and finance, donors and fertiliser suppliers to plan and coordinate quantity, timing, funding of fertiliser import | National Development Planning Commission (NDCP | National Cabinet | MOFA, Donors, Ministry of Finance, National Fertiliser Companies | NGO, District Assembly, Peasant Farmer Association |  |
| **Accountability of District and Regional Directors/ Procurement** | | | | | |
| Monitoring evaluation and tracking system at the region and district level to monitor the quantity of fertiliser entering the district and region – “ESOKO” system | Network of Rural NGO | Regional and District Assembly  (RCC + DA) | Regional Directors of Agriculture, District Chief Executive, Regional/District Fertiliser Input dealers | Farmers/ NGOs |  |
| Power to take action/report the officials found in violation of the policy | Network of Rural NGO | Regional and District  (RCC + DA) | Regional directors of Agriculture, District Chief Executive, Regional/District Fertiliser Input dealers | Regional Security Council, Police |  |
| **Illegal sale of subsidized fertiliser to Burkina Faso and other districts at higher price:** | | | | | |
| Inclusion of farmer representative in fertiliser allocation committee at district level in the allocation of quotas and quantity received for timely dissemination of fertiliser availability | Local NGO | Regional /District Directors of Agriculture, District Chief Executive | Regional /District directors of Agriculture, District Chief Executive  Association of Farmers | Farmer groups/associations, Input dealers, Association of Award Winning Farmers |  |
| **Farmers selling fertiliser subsidy passbook to Dealers because of lack of funds** | | | | | |
| Generating alternate sustainable livelihood options for generating funds for fertiliser purchase - Village saving schemes, Micro Credit from banks – Assistance from Peasant Farmers Association | Network of Rural NGO |  | Regional /District Directors of Agriculture, District Chief Executive | Farmers |  |
| **Low Extension to Farmer Ratio (Challenge for knowledge dissemination)- Not focusing on national policy for extension services but at community level to bridge the extension gap** | | | | | |
| Community based training of farmers (show case)- “Talking Book” tools | NDPC and Community Contact |  | Regional and District Assembly | Farmers |  |
| Motivating volunteering by offering “Best Volunteer Award” through dedicated district budgets | Agriculture Development Bank |  | Regional and District Assembly | Farmers |  |
| Linking national service-Training on agriculture service and deputing in districts/villages | Ministry of Finance |  | NDPC | Farmers |  |
| Training is usually done by projects (NGOs) during projects but not sustainable. Need to institutionalize it through using the formal concept of “Contact Farmer” | Community Contact |  | Regional and District Assembly | Farmers |  |
